# Detection and assessment of Parkinson's disease based on gait analysis: A survey

**DOI:** 10.3389/fnagi.2022.916971

**Published:** 2022-08-03

**Authors:** Yao Guo, Jianxin Yang, Yuxuan Liu, Xun Chen, Guang-Zhong Yang

**Affiliations:** ^1^Institute of Medical Robotics, Shanghai Jiao Tong University, Shanghai, China; ^2^Department of Electronic Engineering and Information Science, University of Science and Technology of China, Hefei, China

**Keywords:** gait analysis, Parkinson's disease, PD detection and staging, FOG event detection and intervention, gait-based intervention

## Abstract

Neurological disorders represent one of the leading causes of disability and mortality in the world. Parkinson's Disease (PD), for example, affecting millions of people worldwide is often manifested as impaired posture and gait. These impairments have been used as a clinical sign for the early detection of PD, as well as an objective index for pervasive monitoring of the PD patients in daily life. This review presents the evidence that demonstrates the relationship between human gait and PD, and illustrates the role of different gait analysis systems based on vision or wearable sensors. It also provides a comprehensive overview of the available automatic recognition systems for the detection and management of PD. The intervening measures for improving gait performance are summarized, in which the smart devices for gait intervention are emphasized. Finally, this review highlights some of the new opportunities in detecting, monitoring, and treating of PD based on gait, which could facilitate the development of objective gait-based biomarkers for personalized support and treatment of PD.

## 1. Introduction

Parkinson's Disease (PD) is a chronic and progressive neuro-disorder that affects movement (Poewe et al., 2017; Armstrong and Okun, 2020). Apart from Alzheimer's Disease, PD is ranked second-most common neurodegenerative disorder that affects 2–3% of the population over 65 years (Dorsey et al., 2018). In terms of pathophysiology, PD is characterized by the loss of dopaminergic neurons in the substantia nigra, leading to a reduced amount of dapamine in the brain. Hence, this will cause reduced capability of movement control, manifesting as slowness and abnormalities in gait (Armstrong and Okun, 2020). The biomarker for PD is α-synuclein protein in the Lewy bodies. When the function of α-synuclein protein is disrupted, Oligomer, i.e., the main component of Lewy bodies, is generated and damages brain cells (Du et al., 2021). Although extensive research has been conducted to determine the underlying mechanism, the explicit relationship between the loss of neurons and PD is still not fully understood.

Thus far, it is believed that PD is an age-related disease and could be raised by a combination of genetic changes and environmental factors. Aging is one of the leading causes of PD, and its prevalence increases with age. The average age of PD patients is about 60 years old, and PD is rare in people under 40 years old (Poewe et al., 2017). With the progression of ages, the degradation of protein metabolisms or mitochondrial functions will potentially lead to cell death of the dopaminergic neurons in the substantia nigra. About 15% of PD patients have a family history, and 5–10% of them have a monogenic form of the disease with Mendelian inheritance. Till now, a number of genetic risks and variants of PD have been found in extensive studies (Deng et al., 2018). In addition, researchers have conducted various studies to explore the relationships between environmental factors and PD, where the incident rate of PD was proven to be correlated to smoking, caffeine intake, and other factors (Hernán et al., 2002). However, the influence of environmental factors on PD has not been clearly identified due to the long-term effect of compounding factors. Recently, Klingelhoefer and Reichmann (2015) proposed a hypothesis that PD starts in the enteric nervous system or the olfactory bulb, spreads *via* rostrocranial transmission to the substantia nigra, and further transmits into the central nervous system.

As no precise diagnostic biomarkers for PD have been discovered, early symptoms and clinical examinations are major diagnostic measures (Armstrong and Okun, 2020). For early symptoms, PD patients are commonly encountering with non-movement symptoms (e.g., sleep disorder and visual deterioration), movement difficulties (e.g., slow movement, tremor, rigidity, impaired posture and gait), and cognitive problems (e.g., depression, anxiety, etc.). Physical examinations assessed by clinical scales or imaging examinations *via* Magnetic Resonance Imaging (MRI) are frequently used (Armstrong and Okun, 2020).

Gait represents a person's walking and running patterns, which can be mediated by complicated brain networks, involving cortical regions that are responsible for motor and cognitive functions. As mentioned above, gait impairments and abnormalities are primary symptoms of PD. In the past decades, gait analysis has become a quantitative tool for analyzing different walking disorders and gait abnormalities caused by musculoskeletal and neurological degradation (di Biase et al., 2020). In terms of movement symptoms, there are three main aspects leading to gait impairments and abnormalities (Mirelman et al., 2019). 1) Tremor: shaking usually begins in the hands or limbs, and happens more frequently when resting. 2) Slowness of movement: patients demonstrate reduced gait speed and step length compared to healthy counterparts. 3) Muscle stiffness: the high tension of muscles results in the increased rigidity of patients' posture, which can further influence the stability during human walking. In addition, non-movement symptoms (e.g., cognition impairment, depression, anxiety) also contribute to abnormal gait patterns (Deligianni et al., 2019). Supported by advanced sensing technologies, gait analysis can be performed from the clinical lab studies to daily living environments (Chen et al., 2016; Kour and Arora, 2019; Sun et al., 2020), providing opportunities for gait-based PD detection, monitoring, and intervention.

This review is to provide a comprehensive overview of the currently available detection, monitoring, and intervention schemes of PD through gait analysis. In Section 2, we first address the brain networks involved in human gait, aiming to clarify the underlying mechanism of gait impairments in PD. Next, the gait cycle and commonly used gait parameters are introduced. Besides, the typical gait impairments of PD patients are summarized. In Section 3, the clinical scales that can be used for PD assessment are introduced. We then summarize the available vision-based and wearable systems for gait analysis. Section 4 reviews the state-of-the-art gait-based PD detection/staging and FOG detection/prediction, including feature extraction, learning-based classification and regression methods, and available benchmark datasets. The gait intervention methods in PD are summarized in Section 5, ranging from pharmacological treatment, electrical stimulation, external cues, to interventions supported by smart devices. We conclude several future trends in PD detection, monitoring, and intervention based on gait in Section 6 followed by a conclusion.

## 2. Gait hypokinesia in PD

### 2.1. Brain networks involved in human gait

In the past decades, research attention has been gained on studying brain activity changes along with human walking (Fukuyama et al., 1997). In this section, we briefly address the brain networks related to gait planning and execution.

#### 2.1.1. Cortical and subcortical brain regions

**Figure 1:**
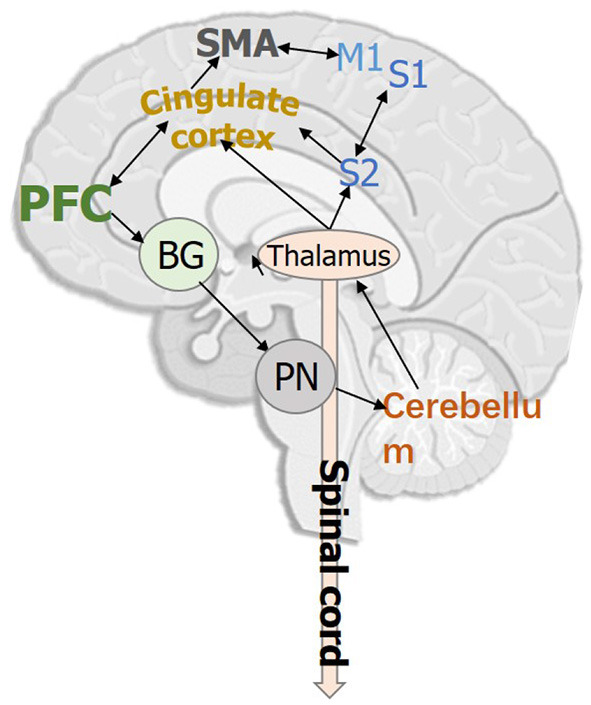
Key cortical and subcortical brain regions that are involved in human bipedal gait. PFC, Prefrontal cortex; M1, Primary motor cortex; S1/S2, Primary/Secondary somatosensory cortex; SMA, Supplementary motor area; BG, Basal ganglia; PN, Pontine nuclei.

Figure 1 demonstrates the key cortical and subcortical regions involved in human gait, which are implicated during human gait. Studies in human neuroscience have proven that the prefrontal cortex, primary/secondary somatosensory cortex, primary motor cortex, supplementary motor area, and the cingulate motor area are highly associated with human gait planning, gait execution, and lower limb movements (Fukuyama et al., 1997; Wei et al., 2022). In addition to the cortical brain regions, several subcortical regions, such as cerebellum, basal ganglia, pontine nuclei, thalamus, form networks also play significant role in regulating human gait and posture (Surgent et al., 2019).

#### 2.1.2. Brain networks for movement

Extensive evidence in human neuroscience supports that basal ganglia is connected to the cerebellum *via* the thalamus and pontine nuclei, where the cerebellum is responsible for maintaining the precision of movement and forms a feedback loop between different cortices (Caligiore et al., 2017). Except for the brain networks related to motor functions, studies have shown that simultaneous cognitive tasks during walking can also affect gait characteristics, which are more pronounced in the elderly and those with neurological conditions (Amboni et al., 2013). Such observations reveal that human gait is influenced by both motor control and human cognition (Lord et al., 2014).

#### 2.1.3. Gait and emotion in PD

There is strong evidence of brain connections between the amygdala and the basal ganglia as well as between the amygdala and the motor cortex (Lagravinese et al., 2018; Deligianni et al., 2019), indicating that there exists a bidirectional interaction between the brain networks of movement and emotion. Especially for PD patients with freezing of gait, brain connectivity between the basal ganglia and the limbic system increased and the connectivity between the basal ganglia and cortical areas decreased (Avanzino et al., 2018). Besides, PD patients also usually show difficulty in recognizing emotions from other people's facial expressions (Lagravinese et al., 2018).

### 2.2. Gait impairments of PD patients

**Figure 2:**
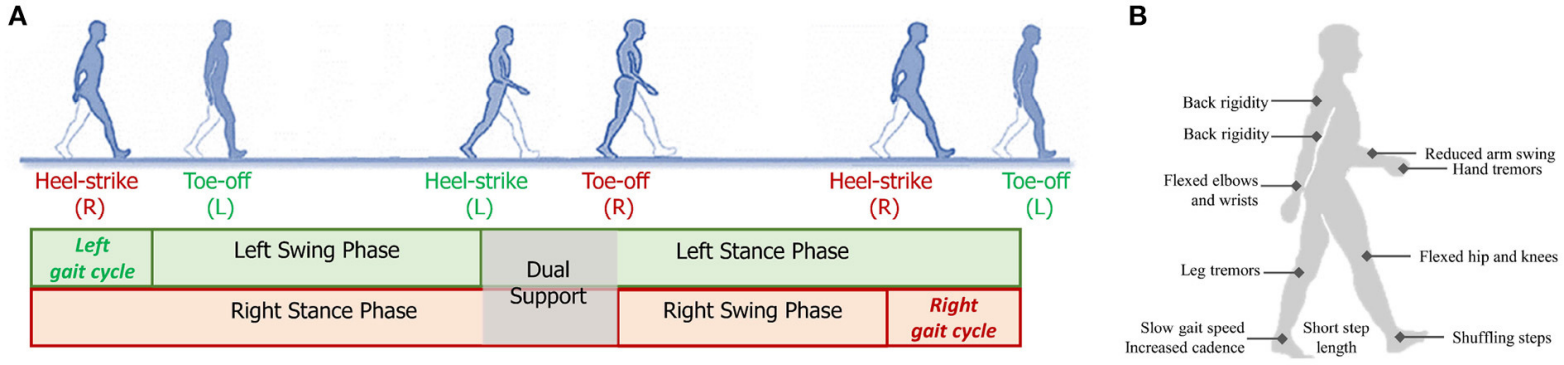
**(A)** Illustration a gait cycle consisting of the swing phase and stance phase; **(B)** Some typical gait and postural symptoms of PD patients.

As PD affects both motor and cognition functions of the brain, the gait patterns of PD patients will demonstrate various impairments and abnormalities, as shown in Figure 2B. We will discuss the gait changes during three different stages, i.e., early, mild-to-moderate, and advanced stages (Mirelman et al., 2019). As shown in Table 1, we summarize several obvious changes in gait parameters that can be used for PD diagnosis. The gait parameters are grouped into three categories, indicating bradykinesia, timing control, and postural stability and gait planning.

**Table 1:** Typical gait parameters and impairments for PD.

**Gait parameters**	**Indications**	**Changes with PD**
Gait speed	Bradykinesia	Reduced
Step/Stride length	Bradykinesia	Reduced
ROM of lower limb joints	Bradykinesia	Reduced
Cadence	Timing control	Increased
Dual support duration	Timing control	Increased
Initiation	Postural stability and Gait planning	Freezing
Turning	Postural stability and Gait planning	Fragmentation
Gait variability and asymmetry	Postural stability and Gait planning	Increased
Limb coordination	Postural stability and Gait planning	Reduced

#### 2.2.1. Early stage

At the early stage of PD, slow gait speed and short step length are first observed (Galna et al., 2015; Pistacchi et al., 2017). However, these gait impairments are not PD-specific signs, as they are age-related and can be induced by many other diseases. Reduced arm swing and movement smoothness, and increased interlimb asymmetry are more specific to PD, which are often unilateral at the early stage (Mirelman et al., 2016; Pistacchi et al., 2017). It is also found that the Range of Motion (ROM) of lower limb joints (i.e., ankle, knee, and hip) becomes smaller, which is more evident during the stance phase (Vallabhajosula et al., 2013). Studies have revealed that the impaired gait patterns become more apparent when PD patients performing dual tasks (Baron et al., 2018).

#### 2.2.2. Mild-to-moderate stage

With the progression of PD, patients will exhibit more severe gait impairments at the mild-to-moderate stage (Mirelman et al., 2019). The risk of falling is increased due to the further instability of posture and gait planning. In specific, shuffling steps, increased dual support and cadence, and reduced arm swing are commonly observed gait changes during this stage (Demonceau et al., 2015; Mirelman et al., 2016). Some patients will demonstrate stooped posture during walking (Mellone et al., 2016) and decompose turning into several fragments (Son et al., 2017).

#### 2.2.3. Advanced stage

For PD patients at the advanced stage, the impairments and abnormalities of gait patterns become even worse. The blocked movement, i.e., Freezing of Gait (FOG), is an episodic yet obvious sign that occurs in most PD patients, which brings a severe burden on patients in daily life (Heremans et al., 2013; Gilat et al., 2021). FOG can be triggered when the patient turns his/her body, traverse narrow corridors, avoid obstacles, and so on. The underlying mechanism for triggering FOG involves a complicated combination of motor, sensory, cognition, and emotion (Nutt et al., 2011; Heremans et al., 2013; Weiss et al., 2020). However, the objective measures and precise biomarkers for FOG still need to be studied. Besides, the balancing, gait planning, and postural stability are gradually reduced, leading to a higher risk of falling (Mirelman et al., 2019). At this stage, some patients will continuously lose motor functions due to the further decline of muscle control, where additional care using wheelchairs or other assistant devices is needed (Creaby and Cole, 2018).

## 3. Gait analysis methodology

### 3.1. Clinical assessment

As shown in the upper part of Table 2, we summarize several common-used observation-based clinical scales and performance-based tests that can be utilized for gait assessment in PD. Some of these scales/tests are PD-specific, including Unified Parkinson's Disease Rating Scale (UPDRS), Hoehn and Yahr (H&Y) Scale, Freezing Of Gait Questionnaire (FOG-Q), and Parkinson's Disease Quality of Life Questionnaire-39 (PDQ-39) (Ebersbach et al., 2006).

**Table 2:** Clinical scales and tests for assessing the gait performance in PD.

**Scales/Tests**	**Scope**	**Descriptions**
UPDRS	Specific	Unified Parkinson's Disease Rating Scale. The most commonly used rating scale for symptoms of Parkinson's disease, covering different aspects of gait
MDS-UPDRS	Specific	A new version of UPDRS modified by Movement Disorder Society
H&Y Scale	Specific	Hoehn and Yahr Scale. Measure how Parkinson's symptoms progress and the level of disability
SAS	Specific	Simpson-Angus Scale. Assess the severity of rigidity and bradykinesia
FOG-Q	Specific	Freezing Of Gait Questionnaire. A widely used tool to quantify FOG severity
PDQ-39	Specific	Parkinson's Disease Quality of Life Questionnaire-39. A self-administered questionnaire containing both motor and non-motor symptoms
10 MWT	General	10 Meter Walking Test. Assess gait speed in a short distance
6-min Walk	General	Assess distance walked over 6 min
TUG	General	Timed Up and Go test. Assess a person's mobility and requires both static and dynamic balance
BBS	General	Berg Balance scale. Assess a person's static and dynamic balance abilities
DGI	General	Dynamic Gait Index. Assess a person's capability of maintain walking balance while performing other tasks

Particularly, UPDRS and H&Y scales are popular in the PD staging tasks, where classification algorithms are developed to predict the severity levels/scales of PD patients from their gait patterns.

The lower part of Table 2 lists several general scales/tests for evaluating gait impairments, which typically measure the gait metrics related to transition, gait, and risk of fall (Toro et al., 2003). These tests/scales can be used as powerful tools for quantifying the gait performance of PD patients after specific gait intervention.

In addition to clinical assessment, as summarized in Table 3, increasing studies leveraged vision-based or wearable sensor based systems to estimate different spatiotemporal, kinematic, and kinetic gait parameters.

**Table 3:** Illustration of different gait analysis systems and their characteristics.

**System**	**Category**	**Pros**	**Cons**	**Selected study**	**Techniques**	**Parameters**
Vision based	Marker based (Mocap)	3D information High accuracy; High freq.; Golden standard	Limited scenario; Cumbersome; Expensive; Tedious setup	Moore et al., 2007	OptiTrack (Mocap)	3D kinematics
				Dillmann et al., 2014	CMS-HS (Mocap)	3D kinematics
				Zhang et al., 2018	Vicon (Mocap)	3D kinematics
				Park et al., 2021	Vicon (Mocap)	Spatiotemporal
	Markerless (camera)	3D Estimation Easy setup; Low cost; Less constraints	Less accurate; Light-sensitive; Data storage; Privacy	Guo et al., 2019	RGBD (Reasense)	2D/3D kinematics
				Eltoukhy et al., 2017	RGBD (Kinect)	3D kinematics
				Ortells et al., 2018	RGB camera	Silhouettes and GEI
				Kidziński et al., 2020	RGB camera	2D kinematics
				Lu et al., 2021	RGB camera	3D kinematics
				Sabo et al., 2022	RGB/RGBD camera	2D/3D kinematics
Wearable sensor based	Pressure insole	Wireless Less constraints; Easy acquisition; Low cost	Uncomfortable; Noisy data; Synchronization Tedious setup Power supply	Alharthi et al., 2020	Force sensors	vertical GRF
				El et al., 2020	Force sensors	vertical GRF
				Marcante et al., 2020	Capacitive pressure	Pressure distribution
				Hu et al., 2021	Capacitive pressure	Pressure distribution
	Inertial ACC IMU			Jarchi et al., 2014	ear-worn IMU	spatiotemporal
				Gonçalves et al., 2021	multiple IMUs	3D kinematics
				Sigcha et al., 2020	waist-worn ACC	3D kinematics
				El-Attar et al., 2021	multiple ACCs	3D kinematics
	EMG			Nieuwboer et al., 2004	surface EMG	Muscle activity
				Volpe et al., 2020	surface EMG	Muscle activity
Platform based	Force	High accuracy; High freq.; Force measurement	Limited scenario; Expensive; Cumbersome	Dyer and Bamberg, 2011	AMTI (Force plate)	COP and GRF
	Optical			Ambrus et al., 2019b	OptoGait	Spatiotemporal
				Ambrus et al., 2019a	OptoGait	Spatiotemporal
Multi modal fusion	Mocap and force plates	Multi-modal gait parameters; Clinical use	Expensive; Cumbersome; Tedious setup	Pereira et al., 2021	Mocap+force plate	Spatiotemporal and 3D kinematics and GRF
				Celik et al., 2022	Mocap+force plate	Spatiotemporal and 3D kinematics and GRF
	Wearable sensor fusion	Same as wearable; Multi-modal gait parameters	Same as wearable; Tedious setup; Low generalization	Negi et al., 2021	IMU+EMG+Insole	Muscle activity and COP, GRF and Spatiotemporal
				Celik et al., 2022	IMU+EMG	Muscle activity and Spatiotemporal
	Vision and Wearable	Multi-modal gait parameters; Robust and accurate	Tedious setup; Low generalization	Gu et al., 2020	Mocap+EMG/ RGBD+EMG	Muscle activity 3D kinematics
				Stack et al., 2018	RGB+IMU	Spatiotemporal and Kinematics

Mocap, Motion Capture System; IMU, Inertial Measurement Unit; EMG, Electromyography; ACC, Accelerometer; GEI, Gait Energy Image; COP, Center of Pressure; GRF, Ground Reaction Force.

### 3.2. Gait parameters

Human bipedal gait involves posture control, balancing, and limb coordination so that the body can move forward in a rhythm (Deligianni et al., 2019; Zanardi et al., 2021). Gait cycle is the critical feature that can be marked by detecting two repetitive gait events, e.g., heel-strike or toe-off, of the same foot. As shown in Figure 2A, a gait cycle can be divided into two phases: stance and swing. Specifically, the gait phase when two feet are contacted to the floor is marked as dual support. Through the use of different gait analysis systems, diverse parameters can be calculated from gait data.

#### 3.2.1. Spatiotemporal parameters

Spatiotemporal parameters refer to the quantitative gait characteristics, which are typically associated with distance (spatial) or time (temporal). These parameters can be calculated based on the extracted gait cycles, such as gait speed, step/stride length, cadence, progression line, walking base width, stance/swing duration, and so on Deligianni et al. (2019) and Zanardi et al. (2021). Among them, stride length is the walking distance of two consensus steps, cadence indicates the steps per minute, and walking base width represents the side-to-side distance between the line of the two heels.

Spatiotemporal parameters are typically be extracted by using vision-based systems and inertial sensors, which have been extensively used for clinical gait analysis as the indication of intrinsic information of the gait patterns (Kour and Arora, 2019). Moreover, these parameters are simple to calculate, allowing analysts to focus on gait analysis rather than parameter extraction.

Previous studies have demonstrated that slow gait speed, short step length, increased cadence and dual support are significant spatiotemporal gait parameters in PD detection and staging (Galna et al., 2015; Caramia et al., 2018; Rehman et al., 2019), which can also be used for validating the effectiveness of gait intervention methods (Schlick et al., 2016; Gómez-Jordana et al., 2018) .

#### 3.2.2. Kinematic gait parameters

In addition to spatiotemporal parameters, the anatomical joints of the human body during walking can be estimated by vision-based systems or multiple IMUs. Along this line, joint positions, joint angles, joint velocities as well as the ROM of each joint, can be derived, which are typically denoted as kinematic gait parameters (Chen et al., 2016; Deligianni et al., 2019). Among different systems, multi-camera Mocap system achieves the highest precision in capturing the human body joints, which is regarded as the golden standard in clnical gait analysis (Moore et al., 2007). Recent advancement in computer vision enables the markerless human pose estimation directly from RGB/RGBD images (Kour and Arora, 2019).

In terms of kinematic gait parameters, studies have shown that PD patients demonstrated decreased ROM of lower limb joints (Vallabhajosula et al., 2013). Human skeletons encodes the walking patterns in a more complicated manner, hence, most of recent studies investigated the use of deep learning models to extract informative features for PD detection and staging (Gu et al., 2020; Lu et al., 2021; Sabo et al., 2022).

#### 3.2.3. Kinetic gait parameters

Kinetic parameters indicate the biomechanics of the human body during walking (Dorschky et al., 2019). The important yet commonly-used kinetic parameters can be measured by force plate or pressure insoles, including foot pressure, Ground Reaction Force (GRF), Center of Pressure (COP), Center of Mass (COM), and joint force/torque. The data collected by surface Electromyography (EMG) reflects muscle activities during walking, which is an alternative way for modeling gait kinetics.

Extensive studies on the PhysioNet dataset has proven that vertical GRF is a critical and discriminative kinetic parameter in the PD detection and staging tasks.

### 3.3. Vision-based gait analysis systems

One of the main methodologies for gait data acquisition is using visual information. As shown in Table 3, we divide vision-based systems into two categories, i.e., marker-based systems for clinical use and markerless systems for home-based environment.

#### 3.3.1. Marker-based system

Multi-camera motion capture (Mocap) is the most common marker-based system, which requires patients to attach reflective markers to their bodies (e.g., the positions related to anatomical joints), then collects the infrared light reflected from the markers passively or actively, and further determines the 3D positions of corresponding markers. Meanwhile, the pre-built model of the human body is constructed maturely to fit the extracted related marker positions. Nowadays, Mocap systems have become the golden standard in clinical gait analysis owing to the high tracking accuracy and sampling frequency (Moore et al., 2007; Zhang et al., 2018; Park et al., 2021). However, such systems consisting of multiple pre-deployed cameras are expensive and cumbersome, limiting the applications to hospitals and labs. Moreover, guidance from specialists and tedious system setup are required. Zhang et al. (2018) utilized Vicon (Vicon, Oxford, UK) Mocap to study the gait performance of PD patients with a motorized walker and proved the effectiveness of the marker-based system in clinical gait analysis.

#### 3.3.2. Markerless system

With the great demands on gait analysis in the household, markerless systems are free from the constraints of tedious setup and wearable markers. Especially with recent advancements of computer vision technologies, 2D/3D human pose (i.e., key joint positions that are similar to Mocap) can be directly inferred from either color or depth images (Shotton et al., 2011; Cao et al., 2017), without the need for pre-build human models. Vision-based markerless systems are more flexible and convenient for pervasive gait monitoring in daily life. However, they can not achieve the accuracy and sampling frequency as the marker-based systems. To overcome the limited sensing area provided by fixed camera, Guo et al. (2019) integrated a single RGBD camera with the mobile robot, and leveraged SLAM to enable the long-term and pervasive 3D gait analysis in a canonical coordinate system. Several studies utilized the 3D skeleton and gait parameters extracted from marker-based systems as the prior to improve the performance of markerless systems. Along this line, Gu et al. (2018) proposed a simple yet effective 3D gait analysis method based on dictionary learning, and Kidziński et al. (2020) developed a deep learning method for enhancing the gait analysis performance.

### 3.4. Wearable sensor-based gait analysis systems

The development of wireless and miniaturized sensors has prospered pervasive sensors-based gait analysis (Chen et al., 2016). Most wearable sensors are inexpensive and portable, which have been widely used in both clinical and home-based scenarios. However, wearable sensing systems are still facing with several inherent challenges, such as uncomfortable to wear, power supply requirements, data synchronization, and noise contamination. In PD studies, research effort has been gained on using different types of wearable sensors to collect real-time spatiotemporal, kinematic, and kinetic parameters.

#### 3.4.1. Pressure/force sensors

Pressure/force sensors are commonly placed in shoes or insoles (e.g., pressure insole), measuring GRF or plantar pressure distribution of the feet when contacting the ground (Marcante et al., 2020; Tahir et al., 2020). GRF can be used to infer the joint force and torque of lower limbs, and the distribution of the foot pressure can also be used to estimate the relevant kinetic gait parameters (e.g., COP and GRF). It should be pointed out that insoles consisting of pressure/force sensors need to be tailored for each individual, avoiding misalignment during walking.

#### 3.4.2. Inertial sensors

The inertial measurement unit (IMU) is one of the most important wearable sensors in gait analysis, which consists of accelerometer, gyroscope, and sometimes magnetometer. By attaching IMUs on the human body, the linear and angular velocity, acceleration, as well as heading reference during gait can be derived, which can be further used for gait event detection and gait stability evaluation (Chen et al., 2016). Previous studies investigated different places for the attachment of IMUs for gait analysis. The most common way is to attach IMU sensors to shoes, ankle joints, knee joints, or the human waist (Mazilu et al., 2013; Caramia et al., 2018; Lee et al., 2018). Jarchi et al. (2014) explored the use of a single ear-worn IMU for gait analysis, and they demonstrated promising results in gait event detection. Recent studies leveraged multiple IMUs, such as Xsens Dot (XSens, Enschede, The Netherlands), attached to lower limbs to recover the 3D skeleton during walking (Gonçalves et al., 2021).

#### 3.4.3. Electromyography sensors

Electromyography (EMG) sensors attached to the skin measure the electrical signals introduced by muscle activities, which can be contaminated by noise originating from cross-talk and motion artifacts (Guo et al., 2021a). Traditional surface EMG systems are inconvenient to set up and constrained in specific scenarios. Recent wireless EMG sensors offer new opportunities for free-living gait analysis and long-term monitoring (Bailey et al., 2018; Steele et al., 2019).

### 3.5. Platform-based gait analysis systems

In terms of platform-based systems for evaluating the gait performance, there are force-based and optical-based platforms according to the sensing mechanism. Force plates are mechanical sensing systems that measure the GRF (both magnitude and direction) during human walking (Halliday et al., 1998). Commonly, force plates are pre-deployed on the floor and the patient will be asked to walk over them. OptoGait (Microgait, Bolzano, Italy) is an optical-based system that using high-resolution technology. By detecting the interruption of infrared beams between transmitter and receiver, OptoGait can acquire accurate gait data of participants (Ambrus et al., 2019a).

### 3.6. Gait analysis *via* multi-modal sensing fusion

To overcome the inherent challenges of each individual system, the combination of multiple gait analysis systems can help obtain more robust and accurate gait parameters. In this review, several popular fusion methodologies are introduced.

#### 3.6.1. Mocap systems and force plates

Recall that marker-based Mocap systems are advantageous in capturing high precision spatiotemporal and kinematic gait parameters, while force plate can measure the kinetic parameters, e.g., GRF. Therefore, the concurrent use of Mocap and force plates becomes popular in clinical gait analysis (Zelik and Honert, 2018). In recent studies, the balance and gait of PD patients were investigated by using the fused systems (Pereira et al., 2021; Ujjan et al., 2022).

#### 3.6.2. Multi-modal wearable sensors

Considering wearable sensors are convenient and portable, a straightforward way is to simultaneously use different wearable sensor systems for gait analysis. With sufficient synchronization, the sensor fusion can overcome the shortcomings of each single modality. For instance, Mazilu et al. (2013) construted the CuPiD dataset by collecting gait data of PD patients with multiple wearable sensors, including IMU sensors attached to different body parts, a smartphone in the pocket, pressure insoles, chest-mounted ECG and head-mounted fNIR. Negi et al. (2021) implemented the fusion of pressure insole, IMU and EMG signals for analyzing different terrain walk. Using IMU and EMG, Celik et al. (2022) introduced a novel data fusion algorithm for enabling gait analysis for both clinical and free-living assessments.

#### 3.6.3. Vision-based and sensor-based wearable sensors

The combination of vision-based and sensor-based systems provides more applicable scenarios by acquiring both the visual and kinematic data. This category can be divided into two aspects according to the way of fusion. One line of research leveraged the Mocap system to evaluate the effectiveness of other wearable sensor-based systems. The others aimed to improve the performance *via* multi-modal fusion against implementing only one system. For instance, Gu et al. (2020) proposed a cross-modal learning method for knowledge transferring between EMG and Mocap (or RGBD and Mocap), thus improving the performance of abnormal gait detection. Stack et al. (2018) exploited both RGB cameras and wearable sensors to enhance the detection of balance impairments in PD.

## 4. Toward automatic recognition in PD based on gait data

**Figure 3:**
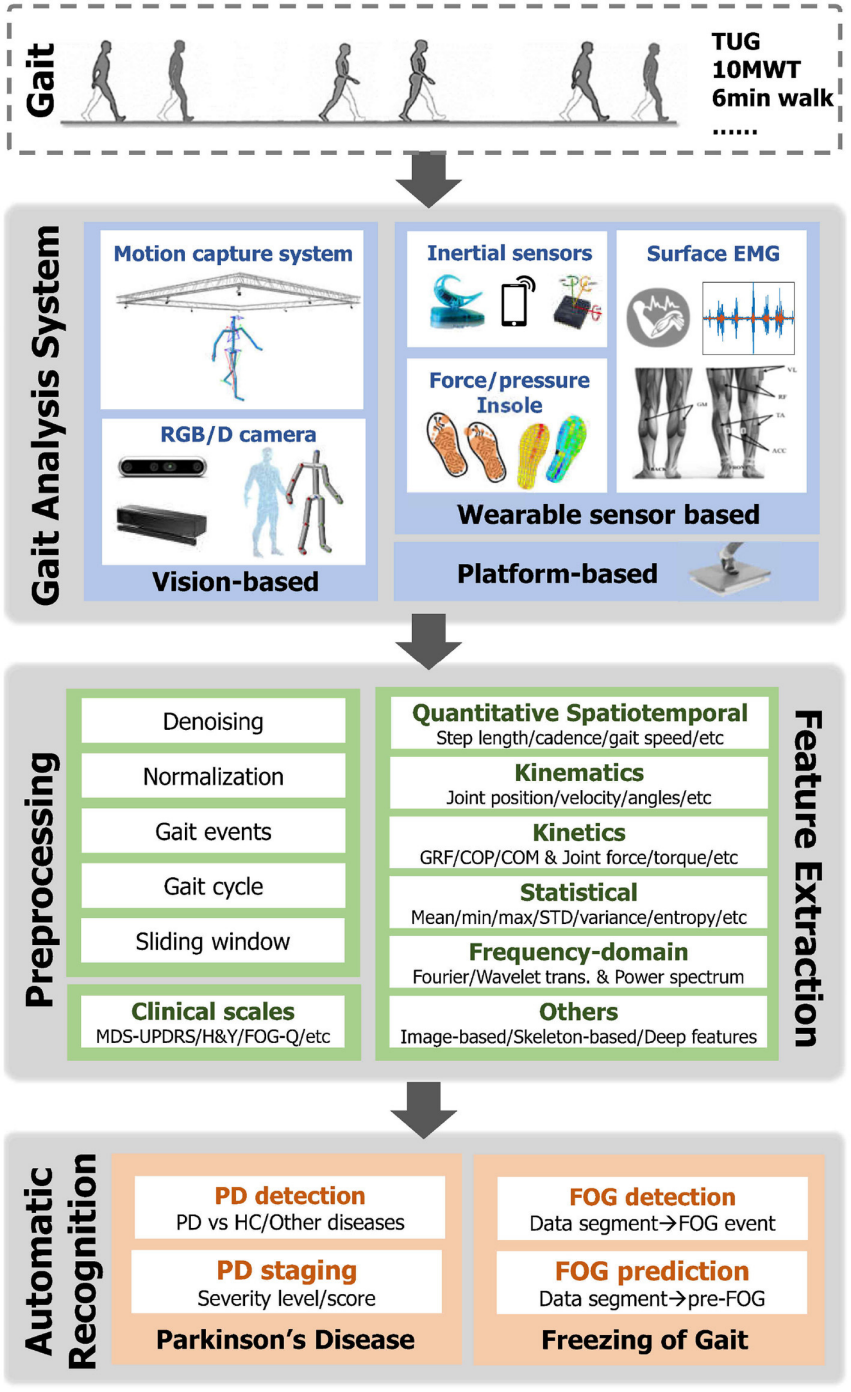
Illustration of the pipeline for automatic recognition in PD based on Gait Data. ROM, Range of Motion; GRF, Ground Reaction Force; COP, Center of Pressure; COM, Center of Mass; trans., transformation; PD, Parkinson's Disease; HC, Healthy Control; FOG, Freeze of Gait.

This section mainly reviews the recent development of gait-based automatic recognition in PD. As illustrated in the lower part of Figure 3, gait analysis has been applied to different tasks, i.e., the detection and staging of PD patients, as well as FOG episodes detection and prediction.

### 4.1. Pipeline overview

An overview of the pipeline of the automatic recognition based on gait data is demonstrated in Figure 3, which typically includes the following steps. **1) Gait data capture**: Human gait can be captured *via* different vision-based and/or wearable sensor-based gait analysis systems as mentioned in Section 3. Meanwhile, clinical scales for assessing the movement and posture stability of PD patients are simultaneously recorded. **2) Data preprocessing**: Given the raw gait data, various preprocessing steps can be first taken. For instance, smoothing and denoising are standard steps to improve the quality of data containing noises and drifts. Another important step is segmenting the time sequence into small fractions, which involves either the gait cycle extraction or the sliding window techniques. Meanwhile, min-max and z-score normalization methods are frequently utilized to remove the bias across segments or trials. **3) Feature extraction**: In order to improve the performance in automatic PD recognition, informative features are additionally extracted from raw gait data. As introduced in Section 3.2, spatiotemporal, kinematic, and kinetic gait parameters are significant features for characterizing walking patterns. To apply these parameters with machine learning models, statistical features and frequency domain features are commonly calculated. Specifically, for the visual input, a number of image-based (e.g., silhouette) and skeleton-based (e.g, key body joints) feature extraction strategies were developed for clinical gait analysis and gait recognition. Recently, advanced deep learning algorithms have gained increasing popularity in gait-based PD detection, providing a unified framework for automatic feature extraction and recognition. **4) Automatic recognition**: Subsequently, the extracted gait features are fed into the dedicated classification models to either recognize PD patients or the occurrence of FOG events (detection) or predict the severity level of PD patients (staging). Intuitively, the detection problem can be treated as a binary classification (i.e., discriminate PD or healthy), while the staging is modeled by regression or multi-class classification. Some early studies designed dedicated rules (e.g., peak detection or thresholding) for classification. However, these rule-based methods are with low generalization capability to new trials and subjects. Lately, extensive Machine Learning (ML) classifiers have been utilized in previous PD detection and staging studies (Mei et al., 2021). Among which the most popular ML models include Support Vector Machine (SVM), Linear Discriminant Analysis (LDA), Random Forest (RF), Decision Tree (DT), K-Nearest Neighbour (KNN), Logistic Regression (LR), AdaBoost, and so on. Recently, with the prosperity of deep learning (DL), deep models have been explored to automatically learn discriminative gait features from different data modalities. **5) Evaluation**: In terms of the evaluation of classification/recognition algorithms, the reported results are validated under either K-fold cross-validation or leave-one-subject-out (LOSO) validation protocols, and the reported metrics include accuracy, precision, recall, F1-score, sensitivity, specificity, and Area Under Curve (AUC).

### 4.2. Gait feature extraction

With the development of wearable sensors and vision-based systems, we have witnessed the flourish of gait analysis in both clinical and healthcare scenarios. However, automatically identifying and staging PD could be more challenging due to the blossom of data modality and capacity (Deligianni et al., 2019; Kour and Arora, 2019). Therefore, the calculation of quantitative gait parameters and the extraction of informative gait features are of paramount importance. In this review, we categorize the existing feature extraction methods into three aspects: **1)** quantitative gait parameters used in clinical gait analysis, **2)** feature extraction from visual inputs, **3)** as well as common features related to wearable sensors.

#### 4.2.1. Quantitative gait parameters

As introduced in Section 3.2, spatiotemporal, kinematics, and kinetics parameters are significant characteristics in describing human walking patterns. The choice of these parameters mainly depends on the data modalities. During the automatic recognition of PD, these parameters can serve as representative features. However, gait abnormalities of PD patients are always coupled with subject-specific characteristics, so conventional gait parameters may be difficult for discriminating subtle gait changes. To extract discriminative gait features and boost the recognition performance in PD, recent effort has been gained on learning-based method for automatic feature extraction.

#### 4.2.2. Feature extraction from vision data

By using the golden-standard Mocap systems, the gait kinematics (e.g., joint positions, velocities, and angles, etc.) as well as spatiotemporal gait parameters (e.g., step length, cadence, gait speed, etc.) can be derived with high precision. Similar to Mocap systems, recent advanced human pose estimation algorithms enable the markerless estimation of 2D/3D key joints of the human body from either RGB or depth images, then the informative gait parameters can be calculated subsequently (Guo et al., 2019; Sabo et al., 2022).

In addition, the key joints of the human body can be modeled as the 2D/3D skeletons connected with links, characterizing both spatial and temporal information of the gait patterns. Previous studies explored extensive methods to extract hand-crafted features from skeletons by distance-based (Guo et al., 2017) and trajectory-based (Guo et al., 2018) mechanisms. For deep learning methods, Recurrent Neural Networks (RNN), showing advantages in processing temporal sequences of diverse length, was first investigated for skeleton-based human motion analysis (Liu et al., 2017). More recently, due to the consideration of spatiotemporal relationship among key joints, Graph Convolutional Networks (GCN) has become the most popular deep models for skeleton-based gait analysis and action recognition (Hu et al., 2019).

In terms of RGB images, the silhouette of the target subject can be extracted and cropped. Conventional methods first reshaped the silhouette images into high-dimensionality silhouette vectors, then aggregated these vectors for PD detection by using statistic methods or spectral transformation (Chen et al., 2012). Gait Energy Image (GEI) extracted from the silhouette is another popular feature for gait-based recognition (Ortells et al., 2018). Similarly, deep learning techniques can extract spatiotemporal features from the RGB video (Guayacán and Mart́ınez, 2021).

#### 4.2.3. Feature extraction from wearable sensing data

The raw temporal sequences collected from wearable inertial, force, pressures, or EMG sensors are first segmented into fractions according to gait cycle detection or pre-defined sliding window, thus the essential gait characteristics can be calculated from each individual segment.

Early works extracted the statistical features from segmented data in the time domain, especially prevalent for EMG data (Guo et al., 2020), including minimum, maximum, mean, median, variance, entropy, etc. Furthermore, the statistical methods, e.g., Principal Component Analysis (PCA) and Linear Discriminant Analysis (LDA), were the most frequently used dimension reduction techniques due to their simplicity and applicability (Demrozi et al., 2019).

Another group of exertions lies in the frequency-domain features. The common-used techniques for transforming original temporal sequences into the frequency domain include Fast Fourier Transformation (FFT), Discrete Wavelet Transformation (DWT), and Continuous Wavelet Transformation (CWT). For instance, the main frequencies and the wavelet coefficients can be regarded as the features (El-Attar et al., 2021). Moreover, the power spectrum, which shows a relationship of decreasing power as a function of frequency, was extensively studied in PD-related research (Capecci et al., 2016). In specific, Freeze Index (FI) is one of the most frequently used acceleration-based features for FOG detection (Moore et al., 2013), which is defined as the ratio of power in the freeze (3–8 Hz) and locomotor (0–3 Hz) bands. It should be noted that FI is specifically designed for detecting the trembling type of FOG.

Nowadays, deep models have become the powerful tools for automatic feature extraction from multi-dimensional sensing data (Rehman et al., 2019). In previous studies, Long Short-Term Memory (LSTM) (Xia et al., 2019) and 1D Convolution Neural Network (CNN) (El et al., 2020) were the most prevalent models due to their advantages in processing temporal sequences.

### 4.3. PD detection and staging based on gait features

Extensive research interests were paid on the development of automatic recognition algorithms based on discriminative gait features, which focused on either recognizing PD patients or estimating the severity level of PD patients from their gait patterns. In Table 4, we conclude several recent works and list them based on the gait capture methodologies.

**Table 4:** Summary of recent studies on automatic detection and staging of Parkinson's Disease.

	**Selected study**	**Subjects**	**Data capture**	**Gait parameters and features**	**Detection algorithm**	**Result**	**Val**.
	Ricciardi et al. (2019)	39PD and 7PSP	Mocap system	Spatiotemporal and kinematics	RF	ACC: 86.4%	10-fold
	Park et al. (2021)	77PD and 34HC	Mocap system	Spatiotemporal and kinematics	RF	ACC: 98.1%	5-fold
	Ajay et al. (2018)	16PD and 13HC	Vision-RGB	Spatiotemporal and kinematics	DT	ACC: 93.8%	10-fold
**PD Detection**	Guayacán and Mart́ınez (2021)	11PD and 11HC	Vision-RGB	Spatiotemporal saliency maps	3D-CNN	ACC: 94.9%	LOSO
	Zhang et al. (2020a)	656PD and 2148HC	IMU (smartphone)	Raw data augmentation	Ensemble of 5 CNNs	AUC: 0.86	5-fold
	Zhao et al. (2018)				LSTM+CNN	ACC: 98.6%	10-fold
	Xia et al. (2019)				CNN+Attn- BiLSTM	ACC: 99.1%	5-fold
	El et al. (2020)				1D-CNN	ACC: 98.7%	10-fold
	Zeng et al. (2019)				RBF-NN	ACC: 98.8%	LOSO
	**Selected study**	**Subjects**	**Data capture**	**Gait parameters and features**	**Detection algorithm**	**Stages**	**Result**	**Val**.
	Lu et al. (2021)	55PD	Vision-RGB	3D human pose	CNN	MDS-UPDRS	ACC: 84.0%	LOSO
	Cao et al. (2021)	18PD	Vision-RGB	Silhouettes	CNN	UPDRS	ACC: 84.2%	3-fold
	Sabo et al. (2022)	53PD	Vision-RGB	2D human pose	GCN	UPDRS SAS	F1: 0.53 F1: 0.40	LOSO
**PD Staging**	Mirelman et al. (2021)	332PD	3-5 IMUs		RUSBoost	H&Y scale	AUC: 0.82	10-fold
	Veeraragavan et al. (2020)				ANN	H&Y scale	ACC: 87.1%	LOSO
	Alharthi et al. (2020)				CNN	H&Y scale	ACC: 95.5%	Hold out
	El et al. (2020)				1D-CNN	UPDRS	ACC: 85.3%	10-fold
	Balaji et al. (2021)				LSTM	UPDRS+H&Y	ACC: 96.6%	Hold out

PD, Parkinson's Disease; HC, Healthy Control; PSP, Progressive Supranuclear Palsy; GRF, Ground Reaction Force; clfs., Classifiers; RF, Random Forest; CNN, Convolution Neural Network; ML, Machine Learning; RBF, Radial Basis Function; Attn, Attention-enhanced; (Bi)-LSTM, (Bidirectional) Long Short-Term Memory; ANN, Artificial Neural Networks; DT, Decision Tree; (MDS)-UDPRS, (Movement Disorder Society)-Unified Parkinson's Disease Rating Scale; SAS, Simpson-Angus Scale; H&Y, Hoehn and Yahr; ACC, Accuracy; AUC, Area Under Curve; F1, F1-score; LOSO, Leave-one-subject-out; Hold out, Random spilit.

#### 4.3.1. PD detection

The PD detection task can be formulated as a binary classification of PD patients and the age-matched healthy controls (HC). Previous works investigated the effectiveness and accuracy of different gait analysis systems and classification algorithms in gait-based PD detection. In addition to this binary detection task, the capability of classifying multiple neurodegenerative diseases was also explored in Wang et al. (2020) and Zhao et al. (2021).

For vision-based systems, Guayacán and Mart́ınez (2021) recently proposed a 3D CNN model that took the spatiotemporal saliency maps of RGB images as input, which achieved 94.1% accuracy (11 PD and 11 HC) under the LOSO validation. In Park et al. (2021), 98.1% detection rate (77 PD and 34 HC) was achieved by using the high precision spatiotemporal and kinematic gait parameters collected by the Mocap system. It can be seen that marker-based systems can provide superior performance in PD detection due the high precision in human skeleton capture, while markerless systems can be deployed in free-living environments.

Inertial sensors were also extensively studied for PD detection. Zhang et al. (2020a) constructed a large-scale dataset (656 PD and 2148 HC) by collecting gait data with a smartphone (i.e., 3-axis accelerometer). Caramia et al. (2018) extracted both spatiotemporal and kinematic gait parameters from 8 IMUs and assembled 6 ML classifier to get a classification accuracy of 96.0%.

For force sensors, the PhysioNet dataset is one of the most popular datasets for PD detection and staging (Goldberger et al., 2000), which contains the vertical GRF data of 93 PD and 73 HC collected by 16 force sensors in insoles. To deal with 16 channels of vertical GRF data, El et al. (2020) directly used 1D-CNN for classification, and Xia et al. (2019) proposed a method by concatenating CNN with an Attention-enhanced Bidirectional LSTM. These methods achieved around 99% recognition accuracy under 10-fold cross-validation. In terms of the more challenging LOSO validation, Zeng et al. (2019) developed the phase space reconstruction and empirical mode decomposition for extracting features from GRF data, where the detection rate was 98% on the PhysioNet dataset.

It can be observed that the PD gait can be well detected from vertical GRF data collected by force sensors. However, it is unfair to directly compare the detection accuracy of different works, as the algorithms were developed based on different datasets/patients, data modalities, and evaluation methods. The inherent challenges in PD detection is that the dataset sizes are usually small-scale, which may impede the development of data-driven deep models. In practice, we may easily collect data from healthy volunteers but having difficulties in PD gait collection, which may introduce the imbalanced data distribution. Such challenges should be tackled in the future research to build accurate and generalized detection models.

#### 4.3.2. PD staging

In addition to the detection of PD from human's gait performance, another line of research aims to predict the severity level of PD patients, which can be formulated as a multi-class recognition problem. As listed in the lower part of Table 4, MDS-UPDRS, SAS, and H&Y are the mostly used clinical scales for rating severity levels of PD patients, which are served as either the labels of training gait data or the ground truths for final validation. To achieve the PD staging task, different machine learning and deep learning models were investigated in previous studies, where gait data could come from wearable sensor-based systems (e.g., force and inertial sensors) or vision-based systems.

In terms of inertial sensors, Caramia et al. (2018) collected gait data from 25 PD patients with 8 IMUs, and extracted spatiotemporal gait features as the input to the different classifiers, where SVM with Radial Basis Function (SVM-RBF) kernel performed best with the accuracy of 75.6%. For staging the patients with H&Y scores, Mirelman et al. (2021) applied a RUSBoost classifier to achieve the accuracy of 82% in PD staging. Due to sensor drift and noise contamination, the classification rates in PD staging with inertial sensors are not satisfactory.

With the recent advancements of computer vision, predicting the severity level from markerless RGB/RGBD cameras has gained increasing attention. In practice, the 2D/3D pose (i.e., key joints) of the target PD patient is first extracted from videos (Sabo et al., 2020, 2022), and then the staging can be performed through dedicated machine learning or deep learning models. In specific, Sabo et al. (2020) extracted both 2D and 3D skeletons of PD patients and then used multivariate ordinal Logistic Regression (LR) models for PD staging. The UPDRS-gait regression models achieved accuracies of 61.4 and 62.1% for 2D and 3D features, respectively. Sabo et al. (2022) leveraged the state-of-the-art SpatioTemporal Graph Convolutional Network (ST-GCN) to predict the PD severity from joint trajectories, which achieved the F1-score of 0.53 and 0.40 for UPDRS and SAS scales, respectively. The significant decrease in model performance compared to Sabo et al. (2020) is due to the individual differences introduced by the LOSO validation, which is more close to the practical scenario. Lu et al. (2020) proposed a model for RGB videos, namely OF-DDNet. The target individuals were first identified and tracked from the recorded RGB videos through time, then an advanced algorithm was applied to extract the corresponding 3D skeleton and body mesh. The proposed OF-DDNet was then used to predict the MDS-UPDRS scores and achieved 84.0% accuracy. Although diverse advanced deep models have been developed for processing visual input, there is still improvement of predicting the severity levels of PD patients.

For the popular PhysioNet dataset, Balaji et al. (2020) utilized four machine learning classifiers to stage PD based on force sensing data, in which DT achieved an accuracy of 99.4% in predicting UPDRS scores. El et al. (2020) and Veeraragavan et al. (2020) took advantage of 1D-CNN and ANN for PD staging, respectively, and achieved similar performance. In order to completely utilize the long-term temporal dependencies in the gait data, Balaji et al. (2021) employed the LSTM model for PD staging, which reached an accuracy of 96.6% on UPDRS and H&Y scores. Similar to the PD prediction task, it can be seen that vertical GRF estimated by force sensors achieved the superior performance in PD staging, compared to other gait parameters and sensing modalities.

### 4.4. FOG event detection and prediction

FOG refers to the sudden and brief episode of inability to produce effective forward stepping (Sun et al., 2020), which is one of the most disabling symptoms of PD patients at advanced stage (Mirelman et al., 2019). Therefore, PD patients with FOG are easily suffered from falling and fall-related injuries (Creaby and Cole, 2018). In recent years, the detection and prediction of FOG events/episodes based on gait data has attracted increasing attention, which can not only facilitate fall prevention but also enable external stimulation for improving FOG.

#### 4.4.1. FOG event detection

In the upper part of Table 5, we summarize recent studies on FOG event detection, i.e., the classification of segmented gait data as FOG or non-FOG episodes. It can be seen that existing works mainly focused on vision-based and inertial sensors (i.e., accelerometer and IMU) for gait data capturing. Previous studies investigated the use of a single inertial sensor. By using the CuPiD database, Mazilu et al. (2016) first explored the effectiveness of using a single wrist-worn IMU for FOG detection, and they found that the wrist-worn setup achieved similar performance as the ankle-worn sensors, achieving the accuracy of 90%. Sigcha et al. (2020) demonstrated the effectiveness of using a single waist-mounted inertial sensor, with 0.923 AUC in FOG event detection. The deployment of multiple inertial sensors was also considered (San-Segundo et al., 2019; Shi et al., 2022), where multiple sensors can be attached to different parts of the lower limb. However, the performance with multiple sensors in FOG event cannot be significantly boosted against the studies using a single inertial sensor.

**Table 5:** Summary of recent studies on detection of FOG event, prediction of FOG event, and discrimination of PD with/without FOG.

	**Selected study**	**Subjects**	**Data collection**	**Gait parameters and features**	**Detection algorithm**	**Result**
	Soltaninejad et al. (2019)	5 PD	Vision-RGBD	Kinematics: foot joint trajectory	Rule-based	ACC: 88.0%
	Hu et al. (2019)	45 PD	Vision-RGB	Kinematics: 2D human pose	GCN	AUC: 0.887
	Cao et al. (2021)	18PD	Vision-RGB	Silhouettes	CNN	ACC: 90.8%
	Ahlrichs et al. (2016)	20 PD	Accelerometer (waist)	Statistical and freq. domain features	SVM	ACC: 95.4%
FOG event detection	Pepa et al. (2020)	44PD	Accelerometer (waist)	Spatiotemporal and freq. domain features	Fuzzy logic	ACC: 93.4%
	Sigcha et al. (2020)	21 PD	Accelerometer (waist)	Freq. domain features	Conv-LSTM	AUC: 0.923
	Camps et al. (2018)	21 PD	IMU (waist)	Freq. domain features	1D-CNN	ACC: 89.0%
	Bikias et al. (2021)	11 PD	IMU (wrist)	Time domain features	CNN	SEN: 83%
	Prateek et al. (2017)	16 PD	IMUs × 2 (heel)	Statistical and freq. domain features	PPF	ACC: 81.0%
	San-Segundo et al. (2019)	10 PD	Accelerometer × 3 (back, thigh, shank)	Freq. domain features	CNN+MLP	AUC: 0.931
	El-Attar et al. (2021)	10 PD		Freq. domain features	ANN	ACC: 93.8%
	Shi et al. (2022)	67 PD	IMU × 2 (ankle)	Freq. domain features and entropy	CNN	F1: 0.92
	Palmerini et al. (2017)	11 PD	Accelerometer × 3 (waist and legs)	Spatiotemporal and freq. domain features	LDA	AUC: 0.76
FOG prediction	Mazilu et al. (2016)	10 PD	Accelerometer × 3 (back, thigh, shank)	Time and freq. domain features	RF	F1: 0.99
	Naghavi and Wade (2019)	10 PD		Freq. domain features	Rule-based	SPE > 85%
	Demrozi et al. (2019)	10 PD		PCA + raw segmented data	KNN	ACC: 94.1%
	(Shalin et al., 2021)	11 PD	Pressure insoles	Kinetics: COP and GRF	LSTM	ACC: 72.5%
	Filtjens et al. (2021)	28 PD	Mocap	Kinematics: 3D human pose	CNN	ACC: 98.7%
FOG vs. nF	Aich et al. (2018)	15nF and 36FOG	Accelerometers × 2 (knees)	PCA + spatiotemporal	4 ML clfs.	ACC: 89.1% (SVM)
	Park et al. (2021)	46nF and 31FOG	Mocap system	Kinematics: 3D human pose	7 ML clfs.	ACC: 98.0% (RF)

PD, Parkinson's Patients; FOG, Freezing of Gait; nF, non-Freezer; Freq., Frequency; GRF, Ground Reaction Force; GCN, Graph Convolution Neural Network; MS-GCN, Multi-stage GCN; GFN, Graph Fusion Network; SVM, Support Vector Machine; CNN, Convolution Neural Network; LSTM, Long Short-Term Memory; PCA, Principal Component Analysis; PPF, Point Process Filter; MLP, Multi-Layer Perception; LDA, Linear Discriminant Analysis; KNN, K-Nearest Neighbour; RF, Random Forest; clfs., Classifiers; ML, Machine Learning; ACC, Accuracy; AUC, Area Under Curve; SPE, Specificity; SEN, Sensitivity; MCC, Matthews Correlation Coefficient.

Recent vision-based works mainly focused on the use of RGB (Cao et al., 2021) or RGBD (Soltaninejad et al., 2019) cameras to detect FOG events in home-based environments. Similarly, most works first extracted 2D/3D key joints directly from visual inputs. Hu et al. (2019) leveraged GCN that takes the human pose sequences as input to predict FOG events during walking. For the video captured from a side view, Cao et al. (2021) extracted the silhouettes of target patients and utilized CNN for classification, achieving 90.4% detection accuracy. With the high-precision human pose collected by the Mocap system, Filtjens et al. (2021) formulated the FOG events detection as a temporal segmentation task from untrimmed skeleton sequences and proposed a Multi-Stage GCN (MS-GCN) method to capture spatial and temporal dependencies. Their method achieved 82.7% accuracy in detecting FOG episodes.

#### 4.4.2. FOG event prediction

In practice, it would be valuable to predict the forthcoming FOG events from the streaming gait data, which can help prevent patients from the potential falling risk or design specific gait intervention techniques. To achieve this goal, most of current studies formulated the prediction as a pre-FOG event detection task (Zhang et al., 2020b), where the time segments before FOG events need to be carefully labeled. Similarly, many previous works investigated FOG prediction based on wearable inertial sensors (Naghavi and Wade, 2019), as listed in the middle part of Table 5. With respect to acceleration data, Zhang et al. (2020b) extracted both spatiotemporal gait parameters and frequency domain features, showing 77% prediction accuracy with the AdaBoost classifier. Demrozi et al. (2019) directly leveraged PCA to select informative raw data segments and reached the accuracy of 94.1% with the conventional KNN classifier. Using pressure insoles, Shalin et al. (2021) extracted COP and GRF and fed them into LSTM to predict pre-FOG events with a successful rate of 72.5%, which is inferior to those using inertial sensors. With the high-precision kinematic features captured by a Mocap system, Filtjens et al. (2021) used a CNN model to precede the FOG episodes, and they proposed layer-wise relevance propagation to enhance the explainability of the deep model, where the pre-FOG events can be successfully detected with a rate of 98.7%. Except for conventional gait parameters, Handojoseno et al. (2014) explored the FOG prediction from EEG signals. They found both power spectral density and wavelet energy could act as biomarkers during FOG.

#### 4.4.3. Freezer detection

In addition to the detection of FOG/pre-FOG events, some other studies aimed to recognize whether a PD patient is a freezer from the gait data. This is similar to the FOG event detection but slightly different, as the freezer detection can be formed as a sequence-level classification, i.e., the PD patient will be marked as the freezer once a single FOG event occurs. In previous works, different ML classifiers were used for achieving this task, as listed in the lower part of Table 5. In specific, Park et al. (2021) achieved 98% accuracy on a dataset with 31 freezers and 46 PD patients without FOG, where high precision 3D gait kinematics were captured by the Mocap system.

It should be noted that current works mainly rely on the accurate annotation of FOG/pre-FOG events of the training gait data, which is a labor-intensive task. Future effort could be gained on the development of unsupervised or semi-supervised methods that can ease the requirements of tedious annotations and facilitate the development of more robust models.

### 4.5. Available datasets

The large-scale dataset is scarce due to not only the complicated collection procedure of conventional gait analysis systems and but also the privacy and ethical issues related to PD patients. Several publicly available datasets raised in recent years are summarized in Table 6.

**Table 6:** Summary of publicly available datasets for gait-based PD research.

**Dataset**	**Subjects**	**Sensors**	**Scales**
Neurodegenerative Gait^†^ Hausdorff et al. (1997)	15 PD, 20 HD, 13 ALS, 16 HC	Force sensor × 4 (insole)	H&Y
PhysioNet (GPD)^†^ Goldberger et al. (2000)	93 PD, 73 HC	Force sensor × 16 (insole)	MDS-UPDRS, H&Y
Smart-Insole^‡^ Chatzaki et al. (2021)	8 PD, 13 HC, 9 Elderly	IMU (feet), Force sensor × 16 (insole)	MDS-UPDRS
CuPiD Mazilu et al. (2013)	18 PD	IMUs × 9, Smartphone pressure insole, ECG(chest)
head-mounted fNIR	-
Daphnet FOG^§^ Bachlin et al. (2009)	10 PD	Acceleromenters × 3 (leg, shank, lower back)	H&Y
mPower^††^ Bot et al. (2016)	1087 PD, 5581 non-PD	IMU (smartphone)	PDQ-8, MDS-UPDRS
Ribeiro De Souza et al. (2022)^‡‡^	35 PD+FOG	Video, IMU	H&Y, FOG-Q, MDS-UPDRS
Kour et al. (2022)^§§^	16 PD	Video (side view), 6 reflective markers	H&Y

^†^ https://physionet.org/content/gaitndd/1.0.0/.

^‡^ https://bmi.hmu.gr/the-smart-insole-dataset/.

^§^ https://archive.ics.uci.edu/ml/datasets/Daphnet+Freezing+of+Gait/.

^††^ https://www.synapse.org/mPower.

^‡‡^ https://doi.org/10.6084/m9.figshare.14984667.

^§§^ https://data.mendeley.com/datasets/44pfnysy89/1.

PD, Parkinson Disease; HD, Huntington's disease; ALS, Amyotrophic Lateral Sclerosis; HC, Healthy Control; FOG, Freezing of Gait; IMU, Inertial Measurement Units; ECG, Electrocardiogram; fNIR, functional near infrared.

Hausdorff et al. (1997) and Goldberger et al. (2000) proposed several wearable sensor-based PD gait datasets in the late 1990s, in which data were collected through multiple force sensors in insoles. Among these datasets, the PhysioNet (GPD) dataset collected from 93 PD patients and 73 healthy control subjects is most commonly used (Kour and Arora, 2019). Along with vertical GRF data, all patients were well annotated based on MDS-UPDRS and H&Y scales, making it easier to benchmark in staging algorithms. Another type of dataset utilized inertial sensors (e.g., accelerometer and IMU) to collect gait data.

The CuPiD database (Mazilu et al., 2013) is a pioneer dataset containing multi-modal sensing data of 18 PD patients, where 11 of them exhibited FOG events during the experiments. The multi-modal data in the CuPiD dataset were collected by 9 IMU sensors attached to different body parts, a smartphone in the pocket, pressure insoles, chest-mounted ECG and head-mounted fNIR. It should be emphasized that many follow-up studies took one or more data modalities to achieve PD detection. The Daphnet FOG dataset (Bachlin et al., 2009) contains data from 10 PD patients collected by three accelerometers, while the mPower dataset (Bot et al., 2016) took advantage of the IMU module in the smartphone and collected data from thousands of participants. Besides, Chatzaki et al. (2021) used both IMU and insole-based force sensors to construct a Smart-Insole dataset.

In addition, the construction of vision-based datasets has attracted increasing attention in recent years. Kour et al. (2022) presented a database recorded side view of 16 PD patients with 6 passive reflective markers attached to the human body. The patients involved are scored with H&Y scale. Ribeiro De Souza et al. (2022), on the contrary, chose to merge the vision-based system with IMU sensors. Walking and turning videos along with the lower limb movements of 35 PD patients were recorded by an RGB camera and a shank-mounted IMU. The H&Y, FOG-Q, and MDS-UPDRS scales were assessed for PD severity levels.

A solid and well-annotated gait database could flourish the development of PD classification and staging algorithms, which should contain a large number of PD patients with clear diagnosis or staging based on clinical scales, as well as the high quality data concentrating on human gait.

## 5. Gait intervention and rehabilitation

**Figure 4:**
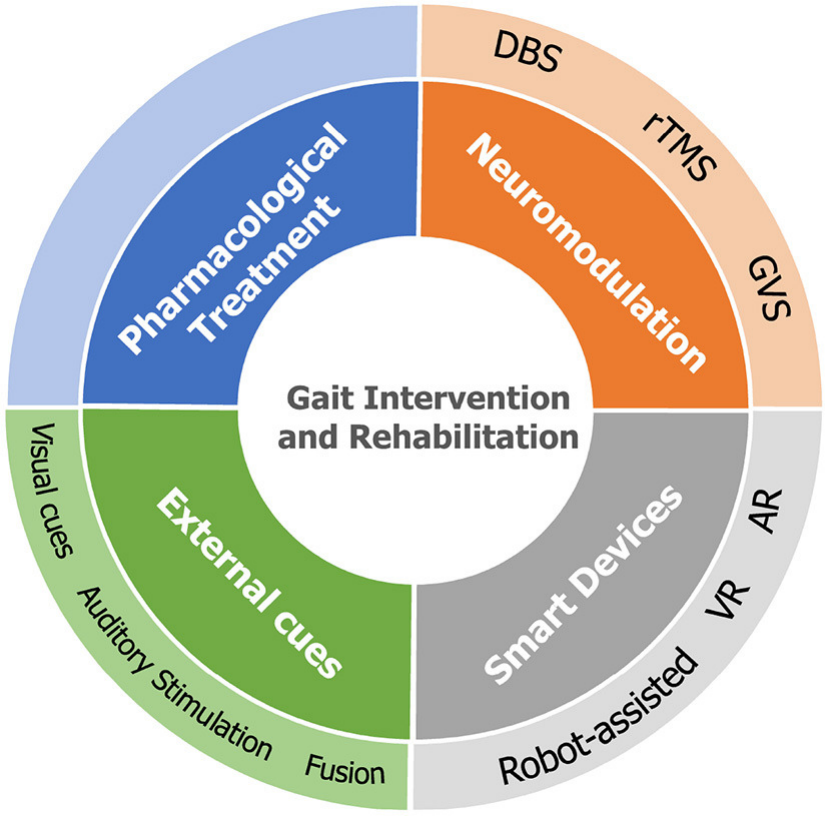
Four categories of gait intervention methodologies in previous studies.

This section concludes the currently available gait intervention and rehabilitation methodologies in previous PD-related studies, as illustrated in Figure 4, which can be categorized into four groups: pharmacological treatment, neuromodulation, external cues, and interventions supported by intelligent devices.

### 5.1. Pharmacological treatment

In the clinical scenario, the most frequently used treatment for gait impairments in PD is dopamine-based treatment, which can help alleviate the motor symptoms (Mirelman et al., 2019; Armstrong and Okun, 2020). In specific, dopamine-based treatments, including Levodopa preparations and dopamine agonists, have been proven to be effective for rigidity, tremor, and disturbance. In specific, gait speed and step length can be improved by using Levodopa, and dopamine agonists can improve the gait initiation and turning movement. It should be pointed out that the pharmacological treatments for non-motor symptoms (e.g., cognitive impairment, depression, and anxiety) can improve gait performance (Connolly and Lang, 2014). More importantly, rehabilitation training and exercise are indispensable complementary to pharmacological treatments.

### 5.2. Neuromodulation

Neuromodulation is the physiological process by using invasive or non-invasive stimulation to regulate diverse populations of neurons.

#### 5.2.1. Deep brain stimulation

DBS is enabled by implanting electrodes into specific areas of the brain, which is considered as an effective invasive intervention for PD. The electrodes can generate electrical pulses to regulate specific cells and chemicals in the brain. Previous studies have demonstrated the efficacy of DBS for alleviating tremors and gait impairments by stimulating the subthalamic nucleus, internal globus pallidus, ventral intermediate nucleus, and pedunculopontine nucleus (Mao et al., 2019). In particular, the high-frequency DBS of the subthalamic nucleus (STN-DBS) is widely used in PD, showing the capability to reduce gait impairments and balance instabilities (Szlufik et al., 2018). Another line of research focuses on the long-term low-frequency STN-DBS, in which the persistent positive effects on FOG and gait variability were observed (Conway et al., 2021). It has been shown that STN-DBS could improve motor functions for up to 10 years, yet the magnitude of improvement tends to decline over time (Limousin and Foltynie, 2019). Studies also suggested that long-term globus pallidus internus (GPi)-DBS had a similar impact on gait impairments as STN-DBS (Mei et al., 2020). Although DBS demonstrated effectiveness in the treatment of PD, it has some disadvantages that makes it less applicable. For instance, the use of invasive electrodes may raise the risk of infection, and some of the patients feel uncomfortable during the treatment.

#### 5.2.2. Repetitive transcranial magnetic stimulation

Compared to the invasive DBS, rTMS is a non-invasive treatment that uses magnetic fields to stimulate neurons in the brain of major depression. In recent years, the capability of rTMS in the treatment of PD has been investigated (Xie et al., 2020). Through the daily rTMS over the primary motor cortex (e.g., foot area), the improvement in walking time was found (Maruo et al., 2013). However, there is no significant improvement on FOG (Dagan et al., 2017) and TUG test (Cohen et al., 2018) found by using rTMS. However, there still lacks evidence-based conversions of rTMS for PD treatment.

#### 5.2.3. Galvanic vestibular stimulation

GVS is a non-invasive brain stimulation method targeting the vestibular system, which aims to improve the balancing and postural instability of PD patients as well as change their gait patterns (Kataoka et al., 2016; Liu et al., 2021b). Liu et al. (2021a) examined the efficacy of GVS for PD patients through the evaluation of network-level connectivity changes. Khoshnam et al. (2018) explored GVS on motor symptoms of upper and lower extremities in PD and found that the variation of the step duration in a TUG test can be improved. Currently, the research on GVS is aimed at various neurorehabilitation applications, while its impact on treating PD patients and improving gait impairment still needs to be studied.

### 5.3. External cues

The use of visual, auditory, and tactile cues has demonstrated effectiveness in improving the gait performance of PD patients, including spatiotemporal gait parameters, FOG, as well as their daily activities.

#### 5.3.1. Visual cues

In past decades, visual cues played an important role in gait intervention for PD patients. The assumptions of the underlying mechanism of visual cueing mainly come from two parts: 1) visual cues, such as the indication of steps, can shift patients' attention to lower limbs, thus compensating the proprioceptive deficit to some extent (Lebold and Almeida, 2011); 2) the synchronization of human gaze behavior and gait patterns (Reed-Jones and Powell, 2017). Stuart et al. (2018) also found that the saccade frequency of PD patients was reduced when walking compared to healthy controls. Their results showed that visual cues could significantly increase the saccade frequency, thus improving PD patients' gait performance. More recently, Stuart et al. (2021) explored the brain activity changes using EEG to demonstrate the effectiveness of visual cues. They found that visual cues could improve the gait performance of PD patients with FOG and simultaneously lead to a larger activity of parietal regions. Table 7 summarizes different types of visual cues explored in previous studies.

**Table 7:** Common-used visual cues and auditory stimulation for PD gait intervention.

**Selected study**	**Visual cues**	**Subjects**	**Gait improvement**
Lebold and Almeida (2011)	Parallel lines (optical flow)	22 PD patients	Increased step length
Vitório et al. (2014)	Parallel lines (white stripes)	19 PD patients	Increased step length
Lee et al. (2012)	Parallel lines (white stripes)	15 PD w/ FOG and 10 PD w/o FOG	Improve gait kinematics significantly of PD with FOG
Schlick et al. (2016)	Footprint	12 PD w/ treadmill	Improved gait speed and stride length
Gómez-Jordana et al. (2018)	Footprint (VR)	12 PD patients	Reduced variation of step length, cadence, and velocity
Barthel et al. (2018)	Laser shoes	21 PD patients	Reduced number and time of FOG
Tang et al. (2019)	Laser cues	34 PD w/ FOG	Improved spatiotemporal parameters and Improved ROM and power generation of ankle/hip joints
**Selected study**	**Auditory**	**Subjects**	**Gait improvement**
Thaut et al. (1996)	RAS @3 rates	15 PD patients	Improved gait velocity, stride length, cadence and timing of EMG patterns
Hausdorff et al. (2007)	RAS @2 rates	29 PD patients	Increased gait speed, stride length, swing time; Reduced variability
Mazilu et al. (2015)	RAS when FOG	5 PD patients	Decreased FoG duration and number
Bailey et al. (2018)	RAS + PT	15 PD patients	Reduced asymmetry of EMG patterns
Erra et al. (2019)	RAS @3 rates	30 PD patients (on and off medication)	Improved GPDI using RAS with 110% of the preferred walking freq
Hove et al. (2012)	Interactive RAS	12 PD patients	Improved fractal scaling to healthy 1/*f* level against fixed-tempo RAS
Pau et al. (2016)	Personalized pace of RAS	26 PD patients	Significant reduction of gait profile score and gait variable score
Ginis et al. (2016)	Verbal feedback	20 PD	Gait and balance improved after 6-week training
Ginis et al. (2017)	4 RAS inputs	15 PD w/ FOG and 13 PD w/o FOG	Freezer showed stable gait under continuous cueing, but preferred intelligent feedback
(Murgia et al., 2018)	Personalized footstep sound and metronome	32 PD patients	Impovements on two RAS groups are equivalent
Marmelat et al. (2020)	RAS w/ fractal step-to-beat	15 PD patients	Synchronize well with fractal RAS with a 1:1 step-to-beat metronome

The number of subjects indicates the one with visual cues or auditory stimulation. VR, Virtual Reality; ROM, Range of Motion; RAS, Rhythmic Auditory Stimulation; GDPI, gait phases quality index; freq, Frequency; FOG, Freezing of Gait; PT, Physical Therapy.

The parallel line attached to the floor was widely used as a visual cue in previous studies, which can regulate the walking patterns of PD patients. Lebold and Almeida (2011) used parallel lines with an interval of 65 *cm*, in the form of optical flow with both normal and reverse directions. They found that the step length of PD patients could be improved regardless of the direction of optical flow. While in the dark environment, the improvement was not significant due to the invisible of lower limbs. Differently, Vitório et al. (2014) observed the improvement of step length under visual step length cues without exproprioception (invisible of lower limbs). They reported that visual cues are critical to the precision of foot placement on targets. Besides, Lee et al. (2012) conducted a more detailed investigation of parallel line visual cues on PD patients w/ and w/o FOG. The results showed that the visual cues had a positive effect, especially for PD with FOG, improving their kinematic gait parameters significantly.

In addition to parallel lines, the virtual footprint display is another critical visual cue, aiming to guide the next steps of the patient. Schlick et al. (2016) used a RehaWalk system to validate the footprint visual cues with treadmill training. After 2 months of training, the gait speed and stride length of PD patients, as well as their performance on the TUG test, were clearly improved. Gómez-Jordana et al. (2018) leveraged Virtual Reality (VR) to display virtual footprint cues in an immersive manner, and found that the variation of step length, cadence, and gait speed were significantly reduced in PD.

Noted that parallel lines and footprints are typically fixed on the floor, limiting the gait intervention to a small area. To overcome this, recent works explored the increase of flexibility by introducing wearable laser cues. Barthel et al. (2018) developed wearable laser shoes, which could automatically project laser cues by detecting heel-strike events. After the study on PD patients with both “off” and “on” medication, the number and lasting time of FOG were significantly reduced. Besides, Amini and Banitsas (2019) used real-time human pose tracking to control a pan/tilt platform to project the laser lines in front of the patients. Tang et al. (2019) conducted a study of laser cues intervention for PD with FOG, providing a comprehensive analysis of the gait spatiotemporal, kinematic, and kinetic changes. The authors reported that spatiotemporal gait parameters, the ROM of the ankle and hip joints, and the power generation of ankle/hip joints were improved *via* the laser cues intervention.

Although studies have been conducted in demonstrating the effectiveness of visual cues, the development of automatic intervention system can be improved by incorporating accurate FOG event detection/prediction modules.

#### 5.3.2. Auditory stimulation

Evidence also reveals that Rhythmic Auditory Stimulation (RAS) can contribute to the improvement of gait and mobility in PD by evoking the brain regions involved in the control of walking (Forte et al., 2021).

Thaut et al. (1996) conducted a pilot study on the effect of RAS on regulating gait patterns of PD patients. After 3-week home-based gait training with rhythmically accentuated music at three tempos, the RAS group demonstrated significant improvement in gait performance and EMG patterns. To deal with different characteristics of PD patients, Hausdorff et al. (2007) set the RAS beat as 100 and 110% of the normal walking rate of each patient, and observed the improvement of gait speed, stride length, and swing time during 100-meter walking. Mazilu et al. (2015) developed a wearable gait training system by giving 8–10 s of audio feedback when the FOG events were detected. They found that four of five PD patients showed decreased FOG duration and FOG number. Pau et al. (2016) leveraged 3D gait analysis to evaluate the effectiveness of RAS on PD patients, and they reported that the gait profile score and gait variable score were significantly reduced after a 3-month follow-up.

More specific, there are many studies that compared different types of RAS in improving PD patients' gait performance. Compared to fixed-tempo RAS, Hove et al. (2012) developed an interactive system using foot sensors to synchronize RAS with human step timing. Results showed that compared to fixed-tempo RAS, the interactive mechanism could increase the fractal scaling to a healthy level. Ginis et al. (2017) compared four input modalities for RAS, i.e., continuous cueing; intelligent cueing; intelligent feedback and no input. In specific, intelligent cueing indicates the beats matched to comfortable cadence, and intelligent feedback is an instruction for users to adapt gait speed. They found that freezers exhibited stable gait under continuous cueing, while non-freezers showed no differences between conditions. The comparison of ecological (personalized footstep sound) and artificial (metronome) RAS were conducted in Murgia et al. (2018), where no difference in gait improvement between the two groups was found. Marmelat et al. (2020) investigated the impact of RAS with different fractal melodic metronomes. Patients with lower persistence benefited better from the fractal ‘metronome' (1:1 step-to-beat ratio) than the 1:2 step-to-beat ratio (‘music'), highlighting the importance of developing patient-specific tests and interventions. In addition to RAS, Ginis et al. (2016) developed a smartphone-delivered gait training system for PD patients performing gait training at home. By detecting gait parameters with wearable inertial sensors, the system provided either positive or corrected verbal feedback while gait parameters remained within or fell outside the therapeutic window. After 6-week training, PD patients showed improved gait and balance compared to the control group. By accurately detecting key gait parameters, it can be concluded from previous studies that personalized RAS could lead to a better improvement of gait performance in PD.

#### 5.3.3. Fused stimulation

An intuitive compound stimulation is the fusion of visual cues and auditory stimulation. Arias and Cudeiro (2008) investigated the external cues in four levels: no cue, auditory cue, visual cue, and auditory-visual cue, where the visual cue was provided by two LEDs integrated into the glasses. They found that auditory stimulation could increase the step length and decrease the stride time, whereas visual stimuli had a negative influence on gait performance. Hence, the fused auditory-visual cue only achieved a similar performance as the auditory stimulation. Lee et al. (2012) suggested that visual cues had positive effects on PD with FOG, while auditory cues more affected gait kinematics in the PD patients without FOG.

Recent research was devoted to exploring the potential of using music and dance as an alternative therapy for PD, where music is a special auditory stimulation (Zhou et al., 2021). By encouraging patients with more exercise, the music and dance therapy can improve cognition, motor control of posture adjustment, and spatial memory, thus having a positive impact on the gait of PD patients as well as their quality of life (de Natale et al., 2017). In specific, Benoit et al. (2014) found that dancing with rhythmic music can improve motor performance, and Hackney and Earhart (2010) reported that the balancing and gait speed were improved after 1-month partnered dance therapy. Among different kinds of dances, Tango dance may preferentially improve the motor and gait performance in PD (McNeely et al., 2015).

### 5.4. Gait intervention by smart devices

#### 5.4.1. Robot-assisted gait training

Research on rehabilitation robotics has gained increasing attention over the last decades due to unmet medical needs for people with neurological and musculoskeletal disorders. Therapeutic robots showing superiority in long-lasting and efficiency can automatically aid patients in providing specific functional movements as well as provide a quantitative assessment of rehabilitation performance. In specific, Robot-Assisted Gait Training (RAGT) received wide attention for helping patients recover from gait impairments induced by stroke, spinal cord injury, cerebral palsy, PD, and Alzheimer's disease (Morone et al., 2017; Alwardat et al., 2018; Fang et al., 2020). Existing therapeutic robots for RAGT can be categorized into end-effector-based, grounded exoskeleton, wearable exoskeleton, and overground gait trainer (or robotic walker) (Hobbs and Artemiadis, 2020; Guo et al., 2021a).

Table 8 summarizes several popular RAGT studies for PD patients. Lo et al. (2010) performed a pilot study to evaluate the effectiveness of RAGT for PD patients with FOG, where four subjects received 30-min sessions of gait training in 10 days. Based on self-report and clinical scales, the improvements in gait speed, stride length, rhythmicity, and limb coordination were observed after RAGT. Barbe et al. (2013) conducted similar research and reported that the FOG of PD patients was improved.

**Table 8:** Summary of robot-assist gait training on PD patients.

**Study**	**Robots**	**Subjects**	**Sessions**	**Gait improvements**
Lo et al. (2010)	Lokomat (GrExo)	4 PD patients w/ FOG	10 × 30min	Improved gait speed, stride length and limb coordination
Barbe et al. (2013)	Lokomat (GrExo)	3 PD patients w/ FOG	10 × 30min	Improved FOG
Kang et al. (2019)	Walkbot-S^TM^ (GrExo)	22 PD patients at H&Y 2.5-3	12 × 45min	Increased gait speed
Yun et al. (2021)	Walkbot-S^TM^ (GrExo)	11 PD patients at H&Y 2.5-3	12 × 45min	Increased gait speed and balancing
Picelli et al. (2012a)	Gait-Trainer (EndEf)	21 PD patients	12 × 45min	Increased gait speed
Picelli et al. (2012b)	Gait-Trainer (EndEf)	17 PD patients at H&Y 3-4	15 × 30min	Increased postural stability
Picelli et al. (2013)	Gait-Trainer (EndEf)	20 PD patients at H&Y 3	12 × 45min	No statistical difference against treadmill training
Picelli et al. (2015)	Gait-Trainer (EndEf)	33 PD patients at H&Y 3	12 × 45min	No statistical difference against balance training
Galli et al. (2016)	G-EO system (EndEf)	25 PD patients	20 × 45min	Improved pelvic obliquity and hip abduction
Capecci et al. (2019)	G-EO system (EndEf)	48 PD patients at H&Y ≥ 2	20 × 45min	Improved FOG
Pilleri et al. (2015)	Overground gait trainer	20 PD patients at H&Y 3-4	15 × 30min	Increased gait speed and postural stability
Kishi et al. (2020)	Wearable Upperlimb exoskeleton	30 PD patients at H&Y 1	Immediately	Increased arm swing, stride length, and gait speed

The number of subjects indicates the one with robot-assisted gait training. GrExo, Grounded exoskeleton; EndEf, End-effector-based robot; H&Y, Hoehn and Yahr.

Researchers from the University of Verona conducted a series of studies using the end-effector-based Gait-Trainer. In Picelli et al. (2012a), the increased gait speed was observed in the RAGT group compared to physical therapy. Besides, they explored the influence of RAGT on PD patients at Hoehn and Yahr stage 3-4, in which 34 subjects were randomly split into two groups (Picelli et al., 2012b). After a 1-month of intervention, the RAGT group showed increased postural stability compared to those with traditional physiotherapy therapy. Furthermore, they recruited 60 PD patients at Hoehn and Yahr stage 3 and divided them into three groups: 20 with RAGT, 20 with treadmill training, and 20 with conventional physical therapy (Picelli et al., 2013). No statistically significant difference was found in gait performance between the RAGT group and treadmill training groups. Picelli et al. (2015) demonstrated that RAGT was not superior to balance training for improving postural instability in PD patients.

Lately, a number of studies also compared the RAGT with treadmill training in PD. After several sessions of RAGT, Galli et al. (2016) reported that the pelvic obliquity and hip abduction of PD patients were significantly improved, Capecci et al. (2019) found that the FOG could be improved after training with the grounded exoskeleton, and Kang et al. (2019) and Yun et al. (2021) reported the improved gait speed of PD patients.

Instead of the fixed robotic systems, Pilleri et al. (2015) utilized the overground gait trainer to guide users to free walk on the ground. After 15 30-min training sessions in 3 weeks, the gait speed and postural stability of PD patients were significantly increased during 10 MWT, TUG and 360 NT tests. Interestingly, Kishi et al. (2020) investigated the use of wearable robots applying interactive rhythmic stimulation on the upper limbs of PD patients, and reported the positive influence on patients' gait performance, including the increased arm swing amplitude, stride length, and gait speed.

Although robot-assisted gait training has demonstrated its effectiveness in PD treatment, the use of grounded exoskeletons, overground gait trainers, and end-effector-based robotic systems are tedious in system setup and preparation, which are limited in hospitals and laboratories. Currently, only a few works investigate PD gait intervention based on lightweight wearable exoskeletons, which have great potential to be used in home-based environments.

#### 5.4.2. VR/AR-assisted gait training

Virtual Reality (VR) and Augmented Reality (AR) technologies are promising tools that can provide immersive/augmented visual feedback to patients. By modulating the visual perception under specific protocols, the underlying mechanisms of gait rehabilitation and intervention of PD patients can be investigated (Zhu et al., 2021). Meanwhile, such intelligent device enhances the engagement of participants while offering a safe and personalized virtual/augmented environment for gait training.

Meanwhile, various paradigms combined with VR technology were investigated. Ehgoetz Martens et al. (2014) used VR to generate the virtual walkway with different heights, which aims to investigate the fear-of-height mechanism. They found that the anxiety during gait could increase the FOG in PD. To overcome the limitation of the treadmill in triggering FOG, Park et al. (2011) developed adaptive treadmill-VR interfaces that could help identify the person-specific FOG trigger. Gómez-Jordana et al. (2018) leveraged VR to study the influence of visual cues (i.e., footprints) in PD gait training. By designing a specific paradigm in VR, Georgiades et al. (2016) investigated the motor initiation and inhibition deficits in PD patients with FOG. Thanks to its flexibility in visual display, VR was also used for inducing visual perturbation during the balancing study (Chiarovano et al., 2017). We refer the readers to Canning et al. (2020) for more details on VR-based gait training in PD.

AR can be viewed as a non-immersive version of VR, enabling the users to see the augmented virtual display along with the real environment. AR-assisted gait training in PD has started for a few decades (Weghorst, 1997). In previous studies, AR was mainly used for providing visual cues to PD patients (Espay et al., 2010). By using wearable AR goggles, Janssen et al. (2017) compared the 3D and 2D visual cues in PD with FOG, and they subsequently investigated the efficacy of AR-based visual cues on turning in place (Janssen et al., 2020).

## 6. Discussions

Although extensive studies have been conducted to either detect/assess PD based on gait analysis or evaluate the effectiveness of different gait intervention methodologies, there are still opportunities and challenges to explore the full capability of these technologies.

### 6.1. Availability of large-scale and multi-modal datasets

In terms of the development of automatic detection and staging of PD/FOG, there are few databases containing a large number of PD patients as summarized in Table 6, especially the patients at different stages. As human gait also encodes the biometric characteristics and gait abnormalities are quite diverse (Gu et al., 2020; Guo et al., 2021b), the data in a small-scale dataset will be inevitably biased in distribution and lack of diversity, impeding the development of robust and powerful deep neural networks in a data-driven manner. Along this line, the model learned from such datasets will have low generalization to novel subjects or unseen abnormal gait styles. In addition, current datasets commonly contain the gait data captured from a single gait analysis system. We believe that the construction of large-scale datasets with multi-modal gait data will be beneficial for the community. However, it is challenging task to complete such a task, as there are a lot of issues related to ethics, privacy protection, data storage, etc. We believe that the community can cooperate and facilitate the data collection from multi-centers.

### 6.2. Pervasive and markerless gait analysis

At the current stage, motion capture system is the most popular and act as the golden-standard methodology for clinical gait analysis, but limiting the applications only in hospitals or laboratories. To achieve pervasive gait analysis in home-based environments, various wearable systems have been developed (Chen et al., 2016), including inertial sensors, pressure insoles, and EMG sensors. However, the setup of wearable sensors typically needs instructions from specialists. In addition, the wearing places would be divergence across subjects and trials, or change during the experiment, leading to a significant challenge in data processing and analysis. In recent years, 3D vision-based ambient intelligence has gained increasing popularity in healthcare (Haque et al., 2020), which can capture sufficient geometry information as well as protect the privacy of the patients. In specific, implicit neural representation based on simultaneous human pose estimation and shape reconstruction has emerged (Yang et al., 2021, 2022), enabling not only the accurate capture of key joints as the Mocap system but also the human garment and appearance information, which can facilitate the development of pervasive and markerless gait analysis.

### 6.3. From motor symptoms to cognitive degradation

As PD is one of the neurological disorders, patients commonly exhibit both degraded physical and cognitive functions. Previous studies have revealed that the emotional states and cognitive load (e.g., dual-task gait test) will also have an immediate impact on gait performance (Deligianni et al., 2019; Raffegeau et al., 2019). Hence, the postural control and gait impairment during the test are influenced by both physical and cognitive functions. However, existing work in this field mainly focused on the detection and monitoring of movement disorders, where the gait performance induced by patients' cognitive states is overlooked. This would influence the accuracy and robustness of the automatic detection algorithms, leading to biases in the predicted results. Therefore, the simultaneous consideration of behavior and cognition characteristics would be valuable in gait-based PD detection and assessment. Through the capture of physiological signals (e.g., brain signals or eye movement), the cognitive load of the target subjects could be estimated. Then, the dedicated models targeting on the disentanglement of these confounding factors could be established, bringing new insights into the detection and assessment of gait impairment.

### 6.4. Subject heterogeneity and label scarcity

Subject heterogeneity and label scarcity are inherent challenges not only in PD gait detection and staging but also in general healthcare scenarios. On the one hand, the abnormal gait patterns are always entangled with subject-specific features, bringing difficulties in discriminating subtle gait changes and generalizing to novel subjects. Hence, to achieve more accurate and generalized PD gait detection and staging, one possible solution is that the subject differences can be regarded as domain shifts Gu et al. (2020) and Guo et al. (2021b), where the gait data of each subject indicates one domain. Thus, the multi-source domain adaptation or domain generalization methods can be utilized for either diminishing or disentangling the subject characteristics from abnormal gait patterns. On the other hand, label scarcity mainly originates from two aspects. 1) Only limited data of certain stages are available, leading to the data distribution as a long-tailed distribution. Thus, the model would be overfitting for those head (majority) classes. 2) Current annotations rely on the clinical scales provided by clinicians, which means that the labels of abnormal gait are far fewer than the normal groups. In order to solve such issues, future work could focus on the loss design, data augmentation or prototype learning strategies to tackle this challenging yet practical problem (Zhang et al., 2021). Besides, unsupervised or semi-supervised learning will bring opportunities to develop robust models.

### 6.5. Gait analysis for treatment evaluation and prediction

With the advanced sensing technologies and learning-based methods, we have witnessed the prosperity of gait analysis as a quantitative and powerful tool for assessing the effectiveness of treatments/interventions in PD. In these studies, well-designed healthy control groups were chosen for comparison, then the statistical analysis was performed to determine whether a specific treatment/intervention can have a positive impact on gait performance. However, such methods only investigated the correlation between gait patterns and specific treatment/intervention, which may learn the spurious associations due to most patients will receive multiple treatment/interventions during long-term rehabilitation. Hence, it would be valuable to explore the effect of each individual treatment/intervention. Within this field, We believe that the combination of well-designed longitudinal studies and dedicated casual inference models (VanderWeele and Hernan, 2013) will promote the development of evaluating and predicting the gait performance of a specific treatment.

### 6.6. Socially assistant robotics and devices

In terms of robot-assisted gait intervention, current works mainly focus on the rehabilitation or intervention of patients' physical functions. As gait impairments of PD patients are also affected by cognitive degradation, it is of paramount significance to explore the techniques that can modulate PD patients' cognitive profiles, thus improving their gait performance. Recently, socially assistant robots or social robots (Breazeal et al., 2016) have emerged in providing either cognitive training or social assistance for patients with neurological disorders. We believe that socially assistant robotics and devices will bring new opportunities for future gait intervention in PD. For instance, the motor symptoms or gait abnormalities could be alleviated if socially assistant robotics and devices can reasonably reduce the patients' mental workload or modulate their cognition states when the FOG event is detected.

### 6.7. Personalized intervention and treatment

Due to the differences of PD patients in biometric characteristics and motor symptoms, each individual will exert diverse impairment in gait patterns; hence, it is of paramount significance to develop effective and personalized treatment plans (Cahan et al., 2019). Hence, the personalized machine learning algorithms are the prerequisite to achieving this goal. First of all, they are desired to extract and model individual characteristics from gait patterns, enabling more accurate detection and assessment. Built upon this, the human-in-the-loop control strategies (Walsh, 2018) can be then developed for wearable robot-assisted or VR/AR-assisted gait training through the bidirectional human-machine interaction. Accordingly, these techniques can be tailored to deliver personalized treatment according to individuals' profile and special rehabilitation needs.

## 7. Conclusion

Parkinson's disease is one of the leading neurodegenerative diseases worldwide. Hence, the detection and monitoring of Parkinson's disease patients are of paramount importance not only in clinical centers but in their household. PD patients with impaired motor function and cognition exhibit various gait impairments in terms of bradykinesia, timing control, and postural stability, leading to degraded quality of life. In recent decades, gait analysis has become a powerful and effective tool for the detection and assessment of PD patients. From vision-based to wearable sensors, diverse gait analysis systems have emerged with their own advantages and disadvantages. Based on different data modalities, this review mainly concludes the dedicated methods for automatic recognition in PD, ranging from PD detection/staging to FOG event detection/prediction. Gait intervention also plays a significant role in PD treatment, in which the interventions supported by rehabilitation robots and VR/AR have recently attracted increasing popularity.

## Author contributions

YG, XC, and GZY designed the review and revised the manuscript critically. YG, JY, YL, and XC performed the literature search and analysis and wrote the paper. YG, JY, and YL analyzed the obtained articles. All authors have read and agreed to the published version of the manuscript.

## Funding

This work was supported by the Science and Technology Commission of Shanghai Municipality under Grant 20DZ2220400, the Foundation of National Facility for Translational Medicine (Shanghai), the Interdisciplinary Program of Shanghai Jiao Tong University under Grant YG2021QN117, the National Natural Science Foundation of China under Grant 61922075, and the USTC Research Funds of the Double First-Class Initiative under Grant KY2100000123.

## Conflict of interest

The authors declare that the research was conducted in the absence of any commercial or financial relationships that could be construed as a potential conflict of interest.

## Publisher's note

All claims expressed in this article are solely those of the authors and do not necessarily represent those of their affiliated organizations, or those of the publisher, the editors and the reviewers. Any product that may be evaluated in this article, or claim that may be made by its manufacturer, is not guaranteed or endorsed by the publisher.
